# Advances in Alphavirus and Flavivirus Research II

**DOI:** 10.3390/v18030313

**Published:** 2026-03-03

**Authors:** Young Chan Kim, Arturo Reyes-Sandoval

**Affiliations:** 1 Division of Structural Biology, Centre for Human Genetics, University of Oxford, Oxford OX3 7BN, UK; 2 Oxford Vaccine Group, Department of Paediatrics, University of Oxford, Oxford OX3 7LE, UK; 3 Instituto Politécnico Nacional (IPN), Av. Luis Enrique Erro s/n., Unidad Adolfo López Mateos, Mexico City 07738, Mexico; arturoreyess@ipn.mx

Newly emerging and re-emerging arthropod-borne viruses (arboviruses) continue to pose a persistent threat to public health. Transmitted mainly by mosquitoes but also by ticks, alphaviruses and flaviviruses can rapidly expand into new regions, with outbreaks being driven by ecological change, travel, and vector adaptation. This Special Issue, ‘Advances in Alphavirus and Flavivirus Research, 2nd Edition’, builds on our previous collection, bringing together 10 original research articles spanning vector competence and transmission, molecular determinants of infection and replication, surveillance and diagnostics, and clinical or translational insights.

Alonso-Palomares et al. address vector biology and transmission by comparing Mayaro virus infection kinetics in Floridian *Aedes aegypti* with those in New World (*Anopheles albimanus*) and Old World (*Anopheles gambiae*) anopheline mosquitoes [1]. They report rapid infection and dissemination in both Anopheles species, as well as the early detection of the virus in the saliva of *An. albimanus*. They also report species-specific impacts on fecundity and reduced lifespan in *An. gambiae*. These findings refine estimates of transmission risk.

At the population level, da Silva Sousa et al. performed a retrospective assessment of arbovirus circulation in Maranhão, north-eastern Brazil (2019 and 2022) [2]. Using RT-qPCR, viral isolation, and sequencing, the authors identified widespread dengue virus detection, primarily DENV-1, alongside additional detections of DENV-2 and re-emergent DENV-3, as well as chikungunya virus and a documented DENV-1/CHIKV co-infection. This emphasises the need for sustained virological and genomic surveillance in endemic settings.

Faye et al. strengthened the diagnostic toolkit by developing a new real-time qRT-PCR assay for Babanki virus, a Sindbis virus subtype reported in Africa [3]. The assay demonstrated 100% specificity and a detection limit of one RNA molecule per reaction. Its diagnostic performance was evaluated using field-collected mosquito pools, which will support future outbreak investigations and entomological surveillance.

Several studies reveal the molecular mechanisms underlying infection. Huerta et al. identified the low-density lipoprotein receptor-related protein 1 (LRP1) as an essential host factor for dengue virus infection [4]. They demonstrate that LRP1 binds to dengue virions via envelope domain III, and that LRP1 depletion markedly reduces the production of infectious virus. This highlights LRP1 as a potential therapeutic target.

Tseng et al. examined how West Nile virus NS5 is recruited to replication organelles at the rough endoplasmic reticulum [5]. Using imaging and biochemical fractionation, they report that NS1 or NS3 contributes to NS5 membrane association and retention, while processed NS5 remains mainly in the cytosol and nucleus. These findings motivate further studies aimed at disrupting NS5 membrane localisation as an antiviral strategy.

Tran et al. explore host restriction and innate immune mechanisms by purifying endoplasmic reticulum membranes from flavivirus-infected cells to identify proteins enriched during infection [6]. Tripartite motif-containing proteins were particularly abundant, and functional studies suggest that TRIM21 and TRIM14 act as restriction factors against Zika and Langat viruses. These proteins operate as interferon-stimulated genes that contribute to type I interferon-mediated antiviral responses.

Garcia et al. focus on the neglected orthoflavivirus Ilhéus virus, establishing an in vitro system to characterise cytopathology, morphology, and morphogenesis [7]. Ultrastructural analyses of infected Vero cells reveal the formation of double-membrane vesicles, convoluted membranes, and vesicular packets that are consistent with the replication-complex structures that have been described for other flaviviruses. This provides a basis for further investigation of ILHV biology.

Baquero-Pérez et al. investigated the epitranscriptomic landscape of chikungunya virus RNA in infected human cells [8]. Using a combination of mass spectrometry and orthogonal sequencing-based analyses, the authors report the presence of enriched inosine signals by mass spectrometry. However, no corresponding inosine sites are detected by Illumina RNA-seq. Furthermore, no alteration in ADAR1 isoform expression is observed. Together, these results highlight the potential and methodological challenges of mapping RNA modifications in viral genomes.

Two studies provide translational perspectives relevant to disease severity and countermeasure design. First, Chamberlain et al. engineered a single amino acid substitution (A533V) in chikungunya virus nsP1 and observed an attenuated phenotype in a C57BL/6 mouse model, with milder disease and more rapid type I interferon induction compared with the wild-type virus [9].

In the case of dengue, Khaing et al. assessed the levels of angiopoietin-like 4 (ANGPTL4) protein in the plasma of adult patients and reported a significant increase during acute infection compared to healthy controls, with levels declining during convalescence [10]. Although no significant differences were observed between severe and non-severe cases in their cohort, the study supports larger, longitudinal evaluations of vascular permeability mediators as candidate biomarkers.

Together, the ten articles in this second edition highlight the breadth of contemporary alphavirus and flavivirus research. They encompass a wide range of topics, including entomological parameters that influence transmission risk and host pathways that govern entry, replication, and immune restriction, as well as surveillance tools, biomarker exploration, and rational attenuation. We hope this collection stimulates further interdisciplinary efforts to anticipate and mitigate the impact of current and future arboviral threats.
